# Evolutionary dynamics of the LTR retrotransposons roo and rooA inferred from twelve complete Drosophila genomes

**DOI:** 10.1186/1471-2148-9-205

**Published:** 2009-08-18

**Authors:** Nicole de la Chaux, Andreas Wagner

**Affiliations:** 1 Department of Biochemistry, University of Zurich, Zurich, Switzerland; 2 The Swiss Institute of Bioinformatics, Basel, Switzerland; 3 The Santa Fe Institute, Santa Fe, NM, USA

## Abstract

**Background:**

*Roo *is the most abundant retrotransposon in the fruit fly *Drosophila melanogaster*. Its evolutionary origins and dynamics are thus of special interest for understanding the evolutionary history of *Drosophila *genome organization. We here study the phylogenetic distribution and evolution of *roo*, and its highly diverged relative *rooA *in 12 completely sequenced genomes of the genus *Drosophila*.

**Results:**

We identify a total of 164 *roo *copies, 57 of which were previously unidentified copies that occur in 9 of the 12 genomes. Additionally we find 66 *rooA *copies in four genomes and remnants of this element in two additional genomes. We further increased the number of elements by searching for individual *roo*/*rooA *sequence domains. Most of our *roo *and *rooA *elements have been recently inserted. Most elements within a genome are highly similar. A comparison of the phylogenetic tree of our *roo *and *rooA *elements shows that the split between *roo *and *rooA *took place early in *Drosophila *evolution. Furthermore there is one incongruency between the species tree and the phylogenetic tree of the *roo *element. This incongruency regards the placement of elements from *D. mojavensis*, which are more closely related to *D. melanogaster *than elements from *D. willistoni*.

**Conclusion:**

Within genomes, the evolutionary dynamics of *roo *and *rooA *range from recent transpositional activity to slow decay and extinction. Among genomes, the balance of phylogenetic evidence, sequence divergence distribution, and the occurrence of solo-LTR elements suggests an origin of *roo/rooA *within the *Drosophila *clade. We discuss the possibility of a horizontal gene transfer of *roo *within this clade.

## Background

Transposable elements (TEs) are DNA sequences that have the ability to replicate within a genome using a variety of mechanisms [1]. They are present in almost all eukaryotic genomes, and they play an important role in genome evolution by creating genetic variation through their mobility. Most new transposable element insertions have a negative effect on the host's fitness through insertions in genes or regulatory regions [2,3], or through ectopic recombination between two copies of an element [4]. For this reason, their transpositial activity is regulated and suppressed by various host encoded mechanisms, like cytosine methylation [5], repeat induced point mutations [6], and expression silencing by RNA interference [7,8]. The number of active copies also decreases due to random mutations, excision, purifying selection, and stochastic loss [2].

**Figure 1:**
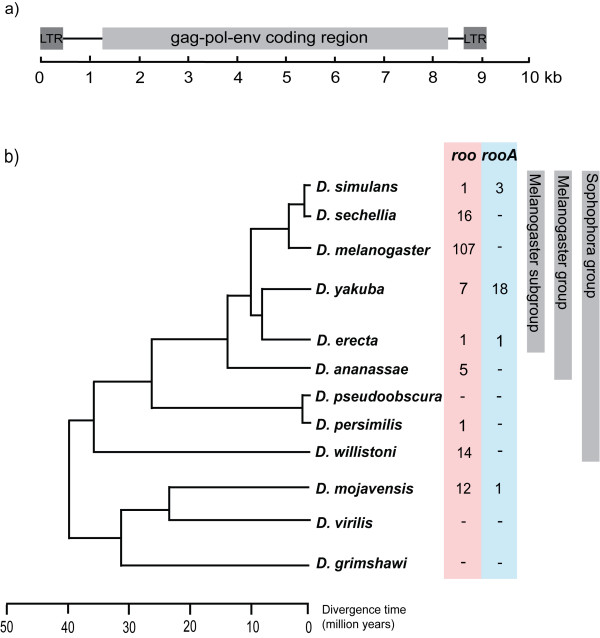
**Roo element structure and phylogenetic tree of the 12 sequenced Drosophila species**. a) The canonical *roo *element is 9092 basepairs (bp) long, has one 7083 bps long ORF (light gray box), and is flanked by 428/429 bps long LTR sequences (dark gray boxes). The ORF encodes the *gag*, *pol *and *env *genes. b) The phylogenetic tree of the *Drosophila *species is taken from [43]. The number of identified *roo *and *rooA *elements is listed for each species. *Roo *and *rooA *elements are highlighted with red and blue bars, respectively.

TEs can be divided into two classes (I and II) based on their replication mechanism. Class I elements use an RNA intermediate for transposition and are called retrotransposons. They can be further subdivided into LTR and non-LTR elements, named after the presence or absence of long terminal repeat (LTR) sequences in the element. Class II elements use a DNA intermediate and are therefore called DNA transposons [1]. Although TEs are mainly transmitted vertically from parent to offspring, they also have the ability to invade new genomes and even cross species boundaries through horizontal gene transfer. In *D. melanogaster *class I and II TEs occupy around 5.35 percent of the euchromatin part of the genome [9], with the LTR element *roo *being the most abundant element [10,11]. *Roo *differs from most other LTR elements in that it encodes an *envelope (env) *gene in addition to the *gag *and *pol *genes encoded by most LTR elements [12]. The origin of *roo*'s *env *gene is not completely clear. It might have been captured from retroviruses, where the gene encodes an envelope glycoprotein that is important for the infectivity of the virus, or it might have been lost from most LTR elements other than *roo*. Aside from *roo*, only a few other LTR elements harbor an *env *protein. With the exception of the *env *gene in the LTR element *gypsy*, the proteins encoded by *env *in LTR retrotransposons are thought to be non-functional. *Roo *was first identified by Scherer et al. under the name *B104 *[13] and by Meyerowitz and Hogness as *roo *[14]. The element is 9092 basepairs (bp) long. It contains one open reading frame (ORF) with 7083 nucleotides that comprise the *gag, pol *and *env *genes. The structure of the element is shown in Figure 1a. The element is strongly expressed during embryogenesis and is responsible for gene mutations [15].

Frequent ectopic recombinations between *roo *elements cause chromosome rearrangements [4]. So far, *roo *has only been identified in a few species belonging to the melanogaster subgroup [16], and one horizontal transfer event between *D. melanogaster *and *D. simulans *has been suggested [17]. An anciently diverged relative of *roo *is *rooA*, which also has members in *D. melanogaster*. In this subfamily, the internal coding region is only ~62 percent identical to the coding region of *roo*. The *rooA *subfamily was probably active in the *D. melanogaster *genome ~2.5 myr ago [18]. In contrast to the high copy number of *roo*, only a few *rooA *elements were previously identified in the *D. melanogaster *genome [11]. These elements are mostly short fragments and up to now, no complete *rooA *element has been identified.

*Roo*'s high abundance makes it a prominent constituent of mobile DNA in *Drosophila*. This fact, and the existence of a diverged subfamily renders the element worthy of study. We here use the 12 sequenced *Drosophila *genomes [9] to study the evolutionary dynamics of *roo *and *rooA *on different timescales. Specifically, we explore these evolutionary dynamics within and among genomes, including likely transmission routes, and we examine the origin of the common ancestor of all *roo/rooA *elements.

## Results

### Distribution of Roo elements

We first used an iterative approach to identify all *roo *and *rooA *elements in the currently available 12 sequenced *Drosophila *genomes [9]. This approach (see Methods for details) used the canonical *Drosophila roo *and *rooA *elements as query sequences to identify a series of additional elements in the *Drosophila *genomes. These additional elements then served as queries in further search rounds, which were continued until no further *roo/rooA *elements could be identified. To identify more diverged elements, we used the 157 high confidence elements identified through this iterative search as initial queries for a second search with less stringent search parameters. Because *roo *and *rooA *query sequences can identify members of both families, we separated all identified elements based on their similarity to the *roo *and *rooA *canonical element. While most elements in *D. mojavensis *correspond to *roo *elements, one element shared a higher similarity to the canonical *rooA *element. All other identified *rooA *elements, however, are much more similar to the *rooA *canonical sequence than this one element. We mention this element, because its dubious affiliation affects the evolutionary scenarios we discuss below.

Our combined search approach (round one and two) yielded a total of 230 elements, 164 *roo *and 66 *rooA *elements, that occurred in nine out of the 12 genomes (see Additional file 1). Columns 2 and 5 of table 1 display the number of *roo *and *rooA *elements, respectively, in each genome, as does Figure 1b in the context of the *Drosophila *phylogeny. Table 1 also lists the number of probably active elements (full-length, no frameshift, no stop-codon). The distribution of elements is different between the two subfamilies. For example, while the *D. melanogaster *genome is the most *roo*-rich genome, harboring 107 elements (two thirds of all identified *roo *elements), it is *D. erecta *that harbors most *rooA *elements (44 copies). Overall, we identified *roo *elements in nine out of the 12 genomes, but found *rooA *elements in only four genomes. We now briefly highlight some features of *roo*'s distribution. The two species most closely related to *D. melanogaster *are the sister species *D. simulans *and *D. sechellia*. In contrast to the large number of *roo *copies in *D. melanogaster*, we find only 16 *roo *copies in *D. sechellia*, and only one fragmented copy in *D. simulans*. We want to note that the genome of *D. sechellia *is not assembled and that the copy number might, therefore, be lower.

**Table 1:** Element distribution and insertion time

	*roo*			*rooA*		
Organism	number of copies/func. copies	solo-LTR	median age in myr	number of copies/func. copies	solo-LTR	median age in myr
*D. simulans*	1/-	102	-	3/-	117	1.18
*D. sechellia*	16/3	113	0.15	-	138	-
*D. melanogaster*	107/50	558	0	-	55	-
*D. yakuba*	7/-	77	0.29	18/-	3335	1.2
*D. erecta*	1/-	47	0.36	44/1	482	0.16
*D. ananassae*	5/1	395	0	-	-	-
*D. pseudoobscura*	-	33	-	-	-	-
*D. persimilis*	1/-	170	1.19	-	-	-
*D. willistoni*	14/-	711	0	-	-	-
*D. mojavensis*	12/1	116	0.09	1/-	35	1.56
*D. virilis*	-	-	-	-	-	-
*D. grimshawi*	-	-	-	-	-	-

total	164/55	2322	0	66/1	4162	0.24

For each genome (column 1 from the left), column 2 shows the total number of identified *roo *elements, with the number of functional copies after the slash. Column 3 shows the number of identified solo-LTRs, which have a similarity of at least 70 percent and a length difference of less than 10 percent to a *roo *LTR sequence. The calculation of the average insertion time (column 4) is based on the divergence between the two LTR sequences of every element. Columns 5–7 represent the same data for the *rooA *element; myr: million years; func: functional;

Like *D. simulans *and *D. melanogaster*, *D. pseudoobscura *and *D. persimilis *are very closely related (Figure 1b). We found no *roo *elements in *D. pseudoobscura*, and only one in *D. persimilis*. This sole element is highly diverged, contains multiple small ORFs as well as a frameshift mutation, and has only 63 percent of the length of the canonical *roo *element. The element may thus be a remnant of a functional *roo *element. The absence of additional *roo *copies in this genome indicates that *roo *is in the process of being eliminated from the genome.

The *roo *copies in *D. mojavensis *deserve special mention. This species is, together with *D. virilis *and *D. grimshawi*, one of the most distantly related species to *D. melanogaster*. The latter two species harbor no *roo *elements, but *D. mojavensis *harbors 12 copies, of which at least one copy may be functional. This tentative assessment is based on the element's complete coding sequence and the absence of frameshifts and stop codons.

With respect to the distribution of *rooA*, we note that the sister species *D. yakuba *and *D. erecta *contain the vast majority of *rooA *elements. Before our analysis, no *rooA *element had been identified outside the *D. melanogaster *genome, and none of the previously identified elements are full length [11]. While none of the elements in *D. yakuba *are full length, *D. erecta *contains at least one full length and probably functional element. Compared to other elements, this element has a 271 amino acid duplication in its coding region, but has only one open reading frame (ORF), and shows no frameshift mutations or internal stop codon. We also note that we did not identify any *rooA *elements in *D. melanogaster*, the species where it was first identified. The flybase annotation of the *D. melanogaster *genome (release 4.1) lists five *rooA *elements. Four of these copies are, however, very short and one element has an extremely diverged coding region, so that our search procedure would not identify it.

### Solo-LTRs indicate loss of roo/rooA in some species

An individual LTR retrotransposon can most easily get lost from a genome via a recombination event between the two LTR repeat units. The remnant of such a recombination event is a solo-LTR, a single remaining LTR repeat unit [19]. Instances of solo-LTRs have been reported for *roo *elements [20]. In order to find out whether some genomes may have lost all their *roo *or *rooA *elements, we searched for *roo *and *rooA *solo-LTR elements in the twelve genomes.

To identify these elements, we performed a stringent blast search [21], using each LTR sequence of all previously identified *roo *and *rooA *elements as queries. We only excluded LTR sequences as queries that were shorter than 200 bp and that contained an "N" (unidentified nucleotide) at more than half of their nucleotides in the genome sequence. This procedure led us to exclude 26 (out of 328) *roo *LTRs and 22 (out of 132) *rooA *LTR sequences as queries. Each identified solo-LTR had to show at least 70 percent identity to the query sequence, and deviate in its length by no more than 10 percent from it. Table 1 (columns 3 and 6) shows the number of identified solo-LTR sequences for each genome.

In addition to solo-LTRs in the genomes that contained *roo *elements, we found 33 solo-LTRs in *D. pseudoobscura*. These observations validate the above assertion that *roo *has become extinct from the genome of *D. pseudoobscura*. A similar extinction process may still be ongoing in the highly mutated sole remaining element of *D. persimilis*.

In contrast to *roo *solo-LTRs, *rooA *solo-LTRs occur only in species within the melanogaster subgroup, with one exception: Solo-LTRs in *D. mojavensis*, which were all identified by the two LTR sequences of the single *rooA *element in this species. The number of *rooA *solo-LTRs varies significantly between the different species in the melanogaster subgroup: Whereas *D. melanogaster *only harbors 55 copies, *D. yakuba *harbors more than 3000 solo-LTRs.

The only two genomes that did not contain any solo-LTRs for either *roo *or *rooA *are *D. virilis *and *D. grimshawi*.

### LTR divergence indicates that most roo elements have been recently inserted

**Figure 2:**
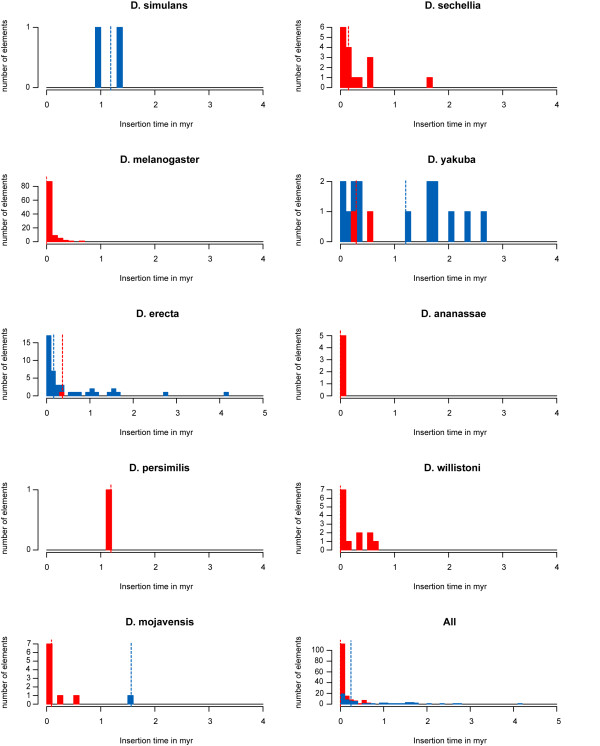
**Insertion time distribution**. The histograms show the estimated insertion time distribution of *roo *and *rooA *(in red and blue bars, respectively), based on the intra-element LTR divergence. Notice the different scales on the y-axis. The vertical red and blue lines indicate the median insertion time in each genome for *roo *and *rooA*, respectively. Two *roo *elements of *D. mojavensis *had to be excluded because their LTR sequences were falsely identified. The histogram in the bottom right corner shows the insertion times of all *roo *and *rooA *elements. myr: million years.

Because the two LTR sequences of a retrotransposon are identical at the time of insertion, their divergence can be used to estimate an element's age, the time since insertion [22]. Highly similar LTR sequences suggest that an element was only recently inserted, because too little time elapsed for many mutations to accumulate between them. From alignments of the LTR sequences of each *roo*/*rooA *element, excluding elements with unidentified nucleotides in their sequences, we estimated the intra-element LTR divergence. We then used this information, together with the synonymous substitution rate of 0.016 substitutions per site per million years (myr) for *Drosophila *[23], to estimate the age distribution of *roo*/*rooA *elements. The median estimated time since insertion for the elements is shown in Table 1 (column 4 and 7 for *roo *and *rooA *elements, respectively) for each genome, and the full age distribution is shown in Figure 2 for *roo *and *rooA *in red and blue bars, respectively. These insertion times have to be considered with caution, because it is possible that transposable elements may experience gene conversion [24,25], which compromise this molecular clock. (For instance, the LTR sequences of *D. ananassae *are identical despite high divergence in their coding regions, a disagreement that might be explained by gene conversion of the LTR sequences.) We, therefore, also show the distance distribution itself (Additional file 2). We want to point out that the insertion time of an element is not the same as the age of the element in the species. Even if all elements in one species appear recently inserted, the element may have resided in the genome for a long time, for example if there is a high turnover of elements within a genome. The high number of solo-LTRs we observe is evidence for such high turnover.

The median age of insertion of all *roo *elements in this analysis is below the detection limit of 0.05 myr obtainable from divergence data, indicating that most *roo *elements were inserted less than a million years ago, as indicated in the bottom right panel of Figure 2. However, there are significant differences in the *roo *age distribution among species. In *D. melanogaster*, the species with most *roo *elements, the vast majority of elements are young, a pattern that is most striking with its more than 100 *roo *elements. Specifically, more than half of the *D. melanogaster *elements have identical LTR sequences, indicating very recent insertion. The median element age is below the detection limit of 0.05 myr. The oldest identified element has inserted only approximately 0.6 myr ago. The *Drosophila melanogaster roo *elements thus underwent many recent transpositions. The median age is even lower than that estimated in previous work [10]. The high LTR identity of the *roo *element is not unusual for LTR elements in *D. melanogaster*. It also occurs for other LTR elements in *Drosophila melanogaster *like *copia*, *blood *or *412 *[10,20]. In *D. melanogaster *the median insertion time for every analyzed LTR element family was less than 0.11 myr ago.

In species other than *D. melanogaster*, the insertion time distributions for *roo *are highly variable. For example, all five *roo *elements from *D. ananassae *have identical LTR sequences. The *roo *elements in *D. sechellia *are very similar to those in *D. melanogaster*, but here only two elements have identical LTR sequences, and the insertion times are more diverse, which suggests that no recent replication activity took place. Finally, in *D. mojavensis *the median *roo *insertion age is 0.09 myr.

The age distribution of *rooA *elements is also quite diverse. *D. erecta*, the species with the highest abundance of 44 *rooA *elements, also shows many young elements but the insertion time distribution is broader than for *roo *in *D. melanogaster*. For example, ten out of the 44 identified *rooA *elements in *D. erecta *have identical LTR sequence, and may have, therefore, been inserted recently, but the oldest element is already approximately 4.1 myr old. The median insertion time, however, is only 0.16 million years ago, which indicates that *rooA *was recently active in this species, and that it might still be spreading. Despite the young age of most elements, the majority of elements may be non-functional, as suggested by the fact that they have only 25 percent of the length of the canonical element, and that some elements also carry stop codons in the coding region. These observations suggest that passive spreading via intact helper elements plays a role in *rooA *proliferation.

In contrast to *D. erecta*, the *rooA *elements in *D. yakuba *have a much higher insertion age (median: 1.02 myr). Most *D. yakuba rooA *elements also have frameshift mutations or stop codons in their coding region. Because *D. yakuba *also shows no signs of recent transposition activity (Figure 2), we speculate that the *rooA *element may be in the process of being deleted from this genome. The putative *rooA *element in *D. mojavensis *is already 1.5 myr old, in contrast to this genome's *roo *elements, which have been recently inserted.

Taken together, the age distribution of *rooA *is broader than that of *roo*.

### Intra- and inter species divergence

We next compared the sequence divergence of different elements within and among species. Such a comparison may yield additional information about the evolutionary dynamics of the *roo*/*rooA *elements. Specifically, we estimated the percent similarity between their protein coding sequences using a maximum likelihood approach implemented in the package Phylip[26]. Because similarity estimation requires coding sequence information, we only used the 134 elements (123 *roo *and 11 *rooA *elements) with a coding sequence length spanning at least half the coding sequence of the canonical element (1180 aa). Most elements with shorter coding sequence are highly diverged and contain several short ORFs interrupted by stop codons and/or frameshift mutations. As a result of this length threshold, we were able to include 10 (of 16) *roo *elements from *D. sechellia*, 97 (of 107) from *D. melanogaster*, 1 (of 7) from *D. yakuba*, all from *D. ananassae*, 3 (of 14) from *D. willistoni *and 7 (of 12) from *D. mojavensis*, and no elements from *D. simulans*, *erecta*, and *D. persimilis*. For *rooA*, we were able to use 2 (of 18) *rooA *elements from *D. yakuba*, and 8 (of 44) elements from *D. erecta*, the single element in *D. mojavensis*.

Tables 2 and 3 show the percent similarity for *roo *and *rooA *elements, respectively, between elements within the same species (diagonal), and among species (off-diagonal). As expected from our LTR analysis, within-species divergence is generally very low, suggesting either recent acquisition or high turnover of *roo *elements within genomes.

**Table 2:** Average protein similarity between roo elements

	*Dsec*	*Dmel*	*Dyak*	*Dana*	*Dwil*	*Dmoj*
*Dsec*	99.1	98	91.9	62.5	61	61.8
*Dmel*		99	91.8	61.9	59	61.3
*Dyak*			100	62.3	60.6	63.2
*Dana*				94.1	49.7	54.4
*Dwil*					93.1	53.9
*Dmoj*						89.8

The 123 *roo *elements with a coding sequence length longer than 1180 aa were used to estimate the amino acid similarity in percent for every pair of these elements using protdist from the PHYLIP package [51]. *Dsec*: *D. sechellia*; *Dmel*: *D. melanogaster*; *Dyak*: *D. yakuba*; *Dana*: *D. ananassae*; *Dwil*: *D. willistoni*; *Dmoj*: *D. mojavensis*

**Table 3:** Average protein similarity between rooA elements

	*Dyak*	*Dere*	*Dmoj*
*Dyak*	98.1	87.6	42
*Dere*		93.8	63.1
*Dmoj*			100

The 11 *rooA *elements with a coding sequence length longer than 1180 aa were used to estimate the amino acid similarity in percent for every pair of these elements using protdist from the PHYLIP package [51]. *Dyak*: *D. yakuba*; *Dere*: *D. erecta*; *Dmoj*: *D. mojavensis*

One conspicuous pattern is that *roo *elements in *D. mojavensis *are 10 percent more similar to elements in *D. melanogaster *than to elements from the more closely related *D. ananassae *and *D. willistoni*. This observation is strictly speaking inconsistent with the *Drosophila *species tree in Figure 1b. However, its weight is reduced if one considers that the similarities between *roo *elements in *D. melanogaster *on one hand, and *D. ananassae*, *D. willistoni *and *D. mojavensis *on the other hand, are of comparable magnitude.

### Phylogenetic incongruence between roo and species tree

**Figure 3:**
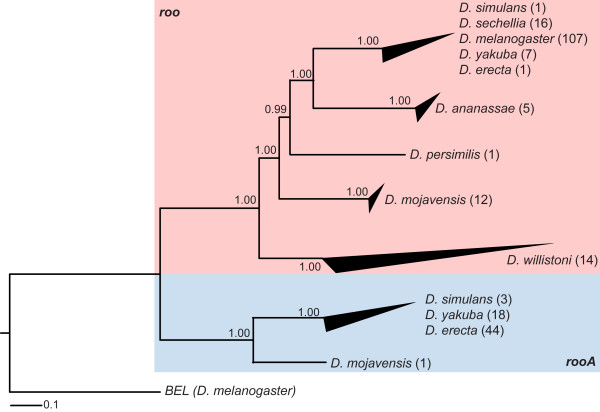
**Phylogenetic tree of the 230 identified roo and rooA elements**. The tree is based on the protein coding sequence of *roo *and *rooA*. It was constructed using PhyML with an approximate likelihood ratio test to estimate the statistical support of the tree topology [27], as shown by the numbers at the branches. All branches have a very high support. There is a clear division between *roo *(red background) and *rooA *(blue background) elements on the tree. Specifically, all *roo *elements from one species form a clade and the same holds for *rooA*. The black triangles at some leaves indicate the divergence of the elements associated with this leaf, with long triangles indicating great divergence. The number in brackets behind each species name indicates how many elements are present in the respective species.

We next constructed and manually inspected a multiple alignment of the protein sequences of *roo*'s and *rooA*'s coding regions. To create a maximum likelihood phylogenetic tree of the *roo*/*rooA *elements from this alignment, we used PhyML_aLRT[27], a version of PhyML[28] that incorporates an approximate likelihood ratio test to estimate the statistical support of the tree topology. Furthermore we used the canonical sequence of the *BEL *element [GenBank:U23420] from *D. melanogaster *as outgroup. The phylogenetic tree of the *roo*/*rooA *elements is shown in Figure 3. Because of the high number of elements the terminal clades are collapsed. The black triangle at the end of each terminal clade indicates the divergence of the elements within a clade or species. Additional file 3 shows the complete tree. *Roo *and *rooA *elements are clearly distinguishable in the tree of Figure 3, because they form two distinct clades (highlighted in red and blue, respectively). Three major patterns are evident from this tree. First, *roo *elements from distantly related genomes are separated in different clades, as one might expect if the mode of transmission is predominantly vertical. Second, elements within one species are usually more closely related to each other than to elements in other species. The only exception are *roo *and *rooA *elements in species of the melanogaster subgroup. In these species, the elements are so closely related that the divergence of the genomes that harbor them is not reflected in the elements' divergence. Third, elements within a genome vary greatly in their divergence. For example, the *roo *elements in *D. mojavensis *and *D. ananassae*, show a low divergence (small triangle), the *roo *elements in *D. willistoni *show the highest divergence (large triangle). *Roo *elements in the melanogaster subgroup also seem to be quite diverged. This divergence can be traced back to only 7 (out of the 132) elements. All other elements are very similar, as can be seen in Additional file 3. High divergence of elements can point to different subclasses of elements, such as functional and non-functional elements.

Despite these general patterns, there is one major incongruency between the species tree (Figure 1b) and the element tree (Figure 3). It regards the *roo *elements. This incongruency occurs in the *D. mojavensis *genome. This species is one of the species most distantly related to *D. melanogaster*. Its common ancestor split from the *Drosophila melanogaster *lineage around 40 myr ago, but the element tree shows that the *roo *elements in *D. mojavensis *are more closely related to *D. melanogaster *than the elements in *D. willistoni*. An error in tree estimation is made unlikely by the high statistical support of both the *D. mojavensis *and *D. willistoni *branches in the *roo *element tree (Figure 3). The observation raises the possibility of a horizontal transfer of the *roo *element into *D. mojavensis *from a *Drosophila *species after the split from *D. willistoni*, but before the split from *D. persimilis*.

### Additional fragments validate our earlier findings

The number of *roo *and *rooA *copies we identified is probably an underestimate of the real copy number in these genomes caused by our high stringency search. We, therefore, performed two additional searches to get a better copy number estimate.

While the coverage and assembly quality of the *D. melanogaster *genome is very good, the quality of most of the other genomes is much lower. Even though the sequence coverage is high, the sequence is divided into many small stretches of contiguous sequence (contigs). The length of many contigs is smaller than 10 kb and therefore smaller than the *roo*/*rooA *element. Identification of complete elements in these genomic regions is, therefore, not possible. Furthermore, the assembly of repeated regions is challenging. To overcome this limitation, we identified the reverse transcriptase (RT), integrase (IN), and protease (PR) domains in the *pol *gene of our high stringency sequences using Pfam domains [29]. The amino acid sequences of these domains, which vary in length from 153 to 210 amino acid, were then taken as queries for a tblastn[21] search. The numbers of identified domains, that do not belong to previously identified *roo *or *rooA *elements, are shown in Table 4.

**Table 4:** Low stringency search and domains

	*roo*					*rooA*				
*Organism*	RT	IN	PR	Total	Trace archive	RT	IN	PR	Total	Trace archive
*D. simulans*	5	4	5	6	+	1	4	1	7	+
*D. sechellia*	2	4	2	20	+	8	1	4	8	+
*D. melanogaster*	8	5	6	115	+	-	1	2	2	+
*D. yakuba*	2	5	5	12	+	21	13	28	46	+
*D. erecta*	2	-	1	3	+	32	25	39	83	+
*D. ananassae*	3	3	6	11	+	-	-	-	-	-
*D. pseudoobscura*	-	3	-	3	NA	-	-	-	-	NA
*D. persimilis*	-	20	-	21	+	-	-	-	-	-
*D. willistoni*	12	12	19	33	+	-	-	-	-	-
*D. mojavensis*	7	5	5	19	+	-	4	-	5	+
*D. virilis*	-	-	-	-	-	-	-	-	-	-
*D. grimshawi*	-	-	-	-	-	-	-	-	-	-

Total	41	61	49	243		62	44	74	151	

For each genome, the number of identified sequence domains, which have a similarity of at least 90 percent and a length difference of less than 1 percent to a reverse transcriptase (RT), integrase (IN) or protease (PR) domain of the identified *roo *and *rooA *sequences, is shown, separated for *roo *(columns 2–4) and *rooA *(columns 7–9). All numbers are additional numbers meaning that domains corresponding to domains in the full length elements were excluded. The number of total elements (column 5 and 10 for *roo *and *rooA*, respectively) was calculated by adding the maximal number of the three domains to the number of elements of the high stringency search. A "+" in column 6 and 11 indicates that a fragment of an *roo *and *rooA *element, respectively, was found in the trace archive for that genome. Note: No trace archive was available for *D. pseudoobscura*

This search identified *roo *domains in all genomes with previously identified *roo *elements, and additional domains in *D. pseudoobscura*, but still did not identify any elements in *D. virilis *and *D. grimshawi*. This leads to the conclusion that the *roo *element is not present in these two genomes, or that it was deleted a long time ago.

The same search identified *rooA *domains only in genomes belonging to the melanogaster subgroup and in *D. mojavensis *(Table 4).

For each of the three domains, our search identified more than 100 domains that were not part of previously identified *roo *or *rooA *elements. We calculated the amino acid similarity between domains within and between elements from different species. The results mainly validate our whole element comparison (see Additional files 4, 5, and 6 for results from the reverse transcriptase, integrase and protease domain, respectively). An exception is the *roo *integrase domain. The *D. pseudoobscura *genome, where we had not identified any element previously, contains a few (three) integrase domains (Table 4). These domains are very similar (92.4% identity) to the domains in the sister species *D. persimilis *(Additional file 5).

Many transposable elements are found in the heterochromatin part of the genome. These regions are usually poorly assembled, which means that we possibly missed some of the *roo *and *rooA *elements. We therefore blasted the amino acid sequence of all *roo *and *rooA *elements against the sequence trace archives of all genomes, with the exception of *D. pseudoobscura*, where no trace archive was available, using a similarity threshold of 70 percent. Table 4 indicates, whether part of a *roo *(column 6) or *rooA *(column 11) element was found. The results are largely confirmatory of our previous analysis. No *roo *or *rooA *elements occur in *D. virilis *or *D. grimshawi*. *Roo *elements occur in the remaining nine genomes. *RooA *elements occur in all genomes of the melanogaster subgroup and in *D. mojavensis*.

### No likely origin of roo/rooA outside Drosophila

To detect if the *roo *or *rooA *element is also present in species outside the *Drosophila *group, we searched for the protein sequence of the canonical element in all 162 available, fully sequenced eukaryotic genomes using genomic blast [30]. We found no complete *roo*/*rooA *elements, and only short fragments covering less than 65 percent of the coding sequence length of *roo*. Specifically, we found similar sequences of length between 1000 and 1540 amino acids that overlapped the complete *pol *region and part of the *gag *regions. The longest of these sequences occurred in insect genomes from mosquitoes *Aedes aegypti*, *Anopheles gambiae*, and *Culex quinquefasciatus*, the silkworm *Bombyx mori*, the parasitoid wasp *Nasonia vitripennis*, and the beetle *Tribolium castaneum*. Sequences of around the same length were also found in the teleost fishes *Danio rerio*, *Gasterosteus aculeatus*, and *Oryzias latipes*, the sea squirt *Ciona savignyi*, the lizard *Anolis carolinensis*, and the sea urchin *Strongylocentrotus purpuratus*. Shorter sequences that still covered the whole *pol *region were found in nematode genomes. No similar sequences were identified in mammals. We next asked whether these sequences correspond to *roo *elements outside the *Drosophila *clade, or if they derive from other retrotransposons which also share *pol *and *gag *regions. To this end, we used our relaxed search procedure (see Methods) on all *roo*/*rooA *elements from *Drosophila*. This relaxed search did not identify any *roo *or *rooA *elements in any of the genomes from the previous paragraph, which suggests that the sequences found by blast stem from other retrotransposon families.

## Discussion

### Element distribution

**Figure 4:**
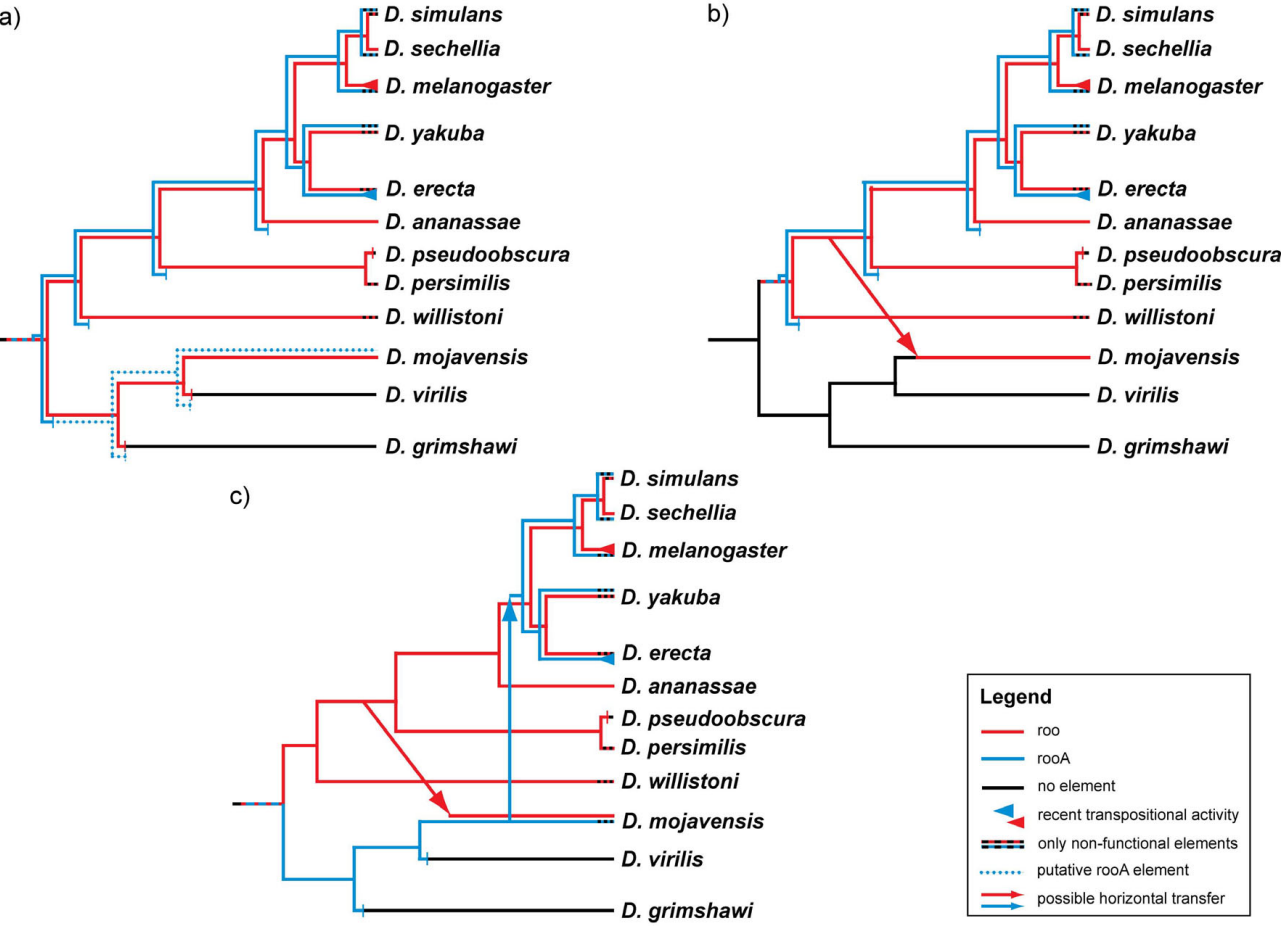
**Three hypotheses for the evolutionary dynamics of the roo and rooA element in the 12 Drosophila genomes**. Red and blue branches indicate species harboring *roo *and *rooA*, respectively. Black branches indicate the loss of both elements. a) *Roo*/*rooA *may have originated in the common ancestor of all 12 *Drosophila *genomes with the split between the two elements shortly thereafter. Both elements were then vertically transmitted, as shown by the red and blue branches. *Roo *was lost in 3 species and *rooA *in six or seven (depending on the correctness of the *rooA *element in *D. mojavensis *(see text for details)). b) *Roo*/*rooA *may have arisen in the common ancestor of the Sophophora group and split shortly thereafter into separate lineages. Both elements were vertically transmitted to all descendant genomes, with a loss of *roo *in one genome and of *rooA *in four genomes. In this scenario, a horizontal transfer of *roo *to the genome of *D. mojavensis *took place after the split from *D. willistoni *(red arrow), but the exact time point of transmission cannot be determined. c) *Roo*/*rooA *might have originated in the common ancestor of all 12 genomes. With the split of the Sophophora group the element evolved into the *roo *element in the Sophophora group, and into the *rooA *element in the other three genomes. While *roo *was vertically transferred to all genomes, *rooA *is today only present in the *D. mojavensis *member of this clade. A horizontal transfer of *roo *to the genome of *D. mojavensis *took place after the split from *D. willistoni*. Later, another horizontal transfer, this time of *rooA*, took place from *D. mojavensis *to the common ancestor of the melanogaster subgroup where it then spread in all five genomes.

We identified 164 copies of the *roo *element in nine out of the 12 sequenced *Drosophila *genomes, and 66 copies of *rooA *in four of the genomes. An additional search for *roo *and *rooA *fragments using sequence domains (see Methods), and a search against the trace archives of the genomes found additional elements. These additional searches identified *roo *elements or remnants of *roo *elements in the genome of *D. pseudoobscura*, where the high stringency search had not found any elements. This observation, together with our observation of solo-LTRs in this species, suggests that *D. pseudoobscura *contains remnants of these elements. In sum, ten of the 12 *Drosophila *species contain *roo *elements. The additional searches also revealed new *rooA *elements or fragments in the two genomes of *D. sechellia *and *D. melanogaster *that did not contain high stringency elements. *RooA *elements are thus present in six out of the 12 genomes, i.e., in the five genomes belonging to the melanogaster subgroup, and in the genome of *D. mojavensis*. Overall, the number of identified elements is probably still an underestimate because not all parts of every genome are sequenced. Especially the high number of genomes which contain only non-functional elements (see Table 1 and Figure 4) is surprising, as it is not likely that these elements were still active recently (as indicated by LTR similarity), and then were lost simultaneously in many genomes. Active elements might still be present in non-sequenced parts of some genomes.

The single *rooA *element in *D. mojavensis *remains a mystery. The solo-LTRs and additional integrase domains we find in this species, were all identified by the sequence of the complete element in this species, and not by any of the element sequences in *D. yakuba *or *D. erecta*. We did not find any traces of *rooA *elements in any other genome outside the melanogaster subgroup. It is possible that this single element belongs to a different LTR element family. Below we will discuss the implications of this element on the evolutionary dynamics of the *roo *and *rooA *families.

### Comparison to previous work

Our analysis can best be assessed by comparison to previous work focusing only on the *D. melanogaster *genome, and to the annotation of the *D. melanogaster *genome. Previously, Sánchez-Gracia et al. (2005) reported 54 *roo *copies based on PCR amplification [17]. This study, however, used only a 1.5 kb fragment of coding DNA to identify a *roo *element. This is much shorter than our thresholds for the high and low stringency search. Vieira et al. (1999) used in situ hybridization to identify *roo *insertion sites in *D. melanogaster *and *D. simulans *[31]. Here again, the probe DNA had to be short (less then 300 bp), which means that no study to date identified complete and active *roo *elements in *D. simulans*. This shortcoming was also pointed out by a recent study by Bartolomé et al. (2009) [32]. Lerat et al. (2003) [20] report 103 *roo *elements, including 30 complete elements. These numbers are similar to the 107 elements we found in *Drosophila melanogaster*. In contrast, Kaminker et al. (2002) [11] report 146 *roo *elements. However, the latter authors identified a sequence as a *roo *element if it was only 50 bp long, a low stringency that is not appropriate for our analysis. At higher stringency, our analysis may be more comprehensive. We note that both previous searches were based on release 3 of the *D. melanogaster *genome, whereas our analysis uses release 4. In release 4, additional gaps in the sequence were closed, adding an additional 1.4 Mb to the genome sequence [33]. The flybase annotation of release 4.1 of the *D. melanogaster *genome still contains 146 *roo *elements. Thirty-six of them were not identified with our search strategy. All but four are truncated elements, i.e., they are solo-LTRs or fragments with a length shorter than our search threshold. Two of the remaining four long elements are not flanked by recognizable LTR sequences; one element has very short LTR sequences; the last element has no identifiable ORF. This comparison shows that our search strategy, together with a search for solo-LTRs and domains, can identify the vast majority of previously known elements. Only short and/or highly diverged element fragments are not identifiable by our search strategy as seen, for example, for the previously identified *rooA *fragments in *D. melanogaster*.

### Roo and rooA are in various states of activity

LTR elements in eukaryotic genomes undergo different waves of activation and inactivation [34,35]. The *roo *and *rooA *elements we observed are in various states of activity. In some species, such as *D. persimilis*, *roo *may be on its way to extinction, as indicated by the sole element in this species, its truncation, its multiple stop codons, its frameshift mutation, and its high divergence. In other species, *roo *elements are numerous and active. Species with abundant *roo *or *rooA *elements have experienced recent transpositional activity. They include *Drosophila melanogaster *for the *roo *element and *D. erecta *for the *rooA *element. In these species, the LTR sequences of any one element are generally very similar to one another, indicating recent insertion. Within-genome amino acid similarity is generally high, also indicating recent transposition. For *D. melanogaster*, these observations are consistent with evidence suggesting that LTR elements have recently become activated [10,36].

The typically young age of most *roo *LTR elements within a genome suggests three possible scenarios for *roo*'s evolutionary dynamics. First, *roo *may have entered any one genome only recently. This possibility apparently contradicts the observation that *roo *elements are moderately diverged within the *Drosophila *clade as a whole, suggesting that they have segregated in the clade for some time. This contradiction could be resolved if *roo *elements that enter a genome cause the host's extinction on short evolutionary time scales, but before they do so, become horizontally transferred to another host. In this case, *roo *elements would persist in a clade largely through horizontal transfer. However, although horizontal transfer has been suggested by Sánchez-Gracia et al. (2005) between *roo *elements from *D. simulans *and *D. melanogaster *[17], and although our data is suggestive of another horizontal transfer event, it contains no indication that such transfer is rampant. For example, the *roo *element tree is largely congruent with the species tree.

The second scenario is that *roo *and *rooA *elements simply show high turnover within a genome, with elements continually becoming deleted (leaving solo-LTRs behind) and transposing into new locations. *D. melanogaster *has indeed a high rate of DNA loss [37]. Compared to other invertebrate genomes, the *D. melanogaster *genome is much smaller and contains less non-coding DNA. A high rate of transposable element deletion may be part of the process maintaining genome compactness. Although most other *Drosophila *genomes are larger than that of *D. melanogaster*, they also have a small genome compared to other invertebrates. The many solo-LTRs we find confirm that the *Drosophila *genomes we study may have a high rate of mobile DNA loss.

A third scenario is that non-autonomous elements are under strong selection to maintain their LTR sequences, because LTR sequences are important for transposition and DNA interaction. Having identical or highly similar LTRs might enable non-autonomous elements to use the transcription mechanism of autonomous copies. This may explain the high coding sequence diversity despite very similar LTR sequences observed for some elements. Examples include the five elements in *D. ananassae*. If this possibility is correct, than many seemingly young elements might not be that young in reality.

In balance, the lack of rampant horizontal transfer, and the frequency with which *roo *elements become excised, support the second and third scenario, which are not mutually exclusive.

### Roo and rooA split a long time ago

We next discuss the relationship of *roo *and *rooA*, whose element trees are clearly separable (Figure 3). The split between *roo *and *rooA *probably took place before the branch leading to *D. willistoni *or even before the branch leading to *D. mojavensis *originated, because otherwise the elements in these genomes would belong to the ancestral *roo/rooA *family and they would be joined to the outgroup in the element tree. The *rooA *family probably consisted of only one or very few copies before the melanogaster subgroup arose, because we did only find one putative occurrence outside this subgroup. The *rooA *copies were probably activated and experienced a transposition activity after the split from *D. ananassae *and then spread through all species, as indicated by the blue lines in Figure 4. One putative *rooA *element, however, occurs in the genome of *D. mojavensis*. This suggests that the split between *roo *and *rooA *indeed occurred in the common ancestor of all 12 species.

### Incongruency between species and roo element tree

We now turn to an incongruency in the subtree for the *roo *elements (Figure 3, red part) that involves the *D. mojavensis *genome. *D. mojavensis *is one of the most distantly related species to *D. melanogaster*, but the *roo *elements in *D. mojavensis *are more closely related to *D. melanogaster *than the elements in *D. melanogaster*'s closer relative *D. willistoni*. The element tree topology (including the branch in question) is well supported, making a tree estimation error unlikely. Incongruencies like these raise the possibility of horizontal gene transfer events, but before one can conclude that such an event took place, a number of potentially confounding factors need to be considered. First, the genomes of *D. willistoni *and *D. saltans*, a sister group of *D. willistoni *with no sequenced genome, are evolving faster than the other genomes of the *Drosophila *genus [38]. This could have influenced the evolution of the *roo *element and explain the position of the *D. willistoni *elements in the element tree. Second, an error in the multiple alignment could lead to this incongruency. Multiple alignments based on many, very long sequences are error prone, especially if the sequences differ in their length and are not all closely related. To exclude this possibility, we examined the average amino acid similarity between elements based on pairwise alignments. This measure of divergence indicates a higher similarity between *roo *elements from *D. mojavensis *and *Drosophila melanogaster *than between elements from *D. willistoni *and *Drosophila melanogaster*. While the difference in similarity is small (61.3 percent versus 59 percent) the result still supports the element tree's structure. A third confounding factor is the species tree itself. The current placement of *D. willistoni *is close to the split of all 12 *Drosophila *genomes; this placement, however, is ambiguous. Many gene trees do not match the current phylogeny [9] and place *D. willistoni *as an outgroup to the other genomes. If so, the species tree would be congruent with the *roo *element tree. In sum, a horizontal gene transfer event involving *D. mojavensis *is possible but does have mixed support in the data.

### High similarity between roo/rooA elements in melanogaster subgroup

Sánchez-Gracia et al. (2005) suggested a horizontal transfer event of *roo *between *D. melanogaster *and *D. simulans *based on synonymous and non-synonymous divergence data. We also find a high similarity in *roo *between these two species. However, we can not unambiguously explain this low divergence by a horizontal transfer event, because the similarity for elements within one genome is higher than the similarity between elements from different genomes. Also, we find high similarity not only between elements in these two genomes but also between all *roo *and *rooA *elements in species of the melanogaster subgroup. This observation might lead to the speculation that the *roo *and the *rooA *element were repeatedly "jumping" from one species to another. However, Sánchez-Gracia et al. showed that other LTR, non-LTR and DNA transposons also show low divergence, raising the possibility that these patterns are consistent with vertical transfer.

### Three possibilities for the evolutionary dynamics of roo and rooA

Taken together, all our observations suggest three alternative hypotheses for the evolutionary dynamics of the *roo *and *rooA *elements among the 12 *Drosophila *genomes. These hypotheses are based on the assumption that the *roo *and *rooA *subfamilies diverged shortly after their common ancestor originated, because they are highly diverged. The first two hypotheses differ in assigning a time for the first occurrence of a *roo*/*rooA *element in the *Drosophila *species. The first hypothesis (Figure 4a) assumes that the *roo*/*rooA *element was already present in the common ancestor of all 12 species, and that both the *roo *and the *rooA *element were vertically transferred to all species. This would explain the absence of *roo *elements in some species by stochastic loss (black branches in Figure 4). Wherever element numbers are low in a genome, such stochastic loss is likely. The closest relatives of genomes with no *roo *elements have small to modest element numbers, which supports the loss hypothesis. For example, the closest relative of *D. pseudoobscura*, which has no *roo *elements, is *D. persimilis*, which has only one *roo *element. According to this hypothesis *roo *was lost from the genomes of *D. virilis *and *D. grimshawi*, but is still present in *D. mojavensis*. The split between *roo *and *rooA *also took place in the common ancestor of all 12 genomes, and *rooA *was transmitted vertically to all genomes. It might have been lost from the common ancestor of the three species outside the Sophophora group soon thereafter. Alternatively, if the single putative *rooA *element in *D. mojavensis *is a true *rooA *element, it might have been lost in each non-Sophophora genome separately (as indicated by the dashed blue lines in Figure 4a).

The second hypothesis addresses the key shortcoming of the first hypothesis, which cannot explain the incongruence between the species (Figure 1b) and the element tree (Figure 3). This second hypothesis places the first occurrence of the *roo*/*rooA *element in the ancestor of the Sophophora group and the split between the two elements shortly thereafter, as shown in Figure 4b by the red and blue branches. The elements were then vertically transmitted to all descendant species. While *roo *elements are still present in all of the nine genomes, *rooA *elements are only found in species belonging to the melanogaster subgroup and were lost from the other genomes. This hypothesis also assumes that the single *rooA *element in *D. mojavensis *belongs to a different LTR family and was wrongly identified. The *roo *element in this genome, however, is explained by a horizontal transfer of a *roo *element after the split of *D. willistoni *to the genome of *D. mojavensis*. This hypothesis can also explain the element tree in Figure 3 (if we assume that the species tree in Figure 1 is correct), the lack of solo LTRs and domains in *D. virilis *and *D. grimshawi*, and the amino acid similarity between the coding regions of the elements. This transfer would have been an ancient transfer, because the elements from *D. mojavensis *share little similarity with elements from other species. An origin of *roo *within the *Drosophila *clade is further supported by the fact that no *roo *elements can be found outside *Drosophila*. Some of the *RETRO *elements in *A. gambiae *show a similarity to the *roo *element [39] but they may not belong to the same family. Furthermore, only LTR sequences of *RETRO *elements have been identified so far, which makes a comparison to the *roo*/*rooA *elements difficult. Previous phylogenetic studies identified the *Tinker *and *BEL *elements in *D. melanogaster *as closest relative of the *roo *element [12,40], but when the split between these elements may have occurred is unclear.

This second hypothesis however, does not explain the single *rooA *element in *D. mojavensis*, if we assume it was not falsely identified. Our third hypothesis (Figure 4c) addresses this shortcoming. As in the first hypothesis, we assume that the *roo*/*rooA *element arose in the common ancestor of all 12 genomes. In contrast to the first hypothesis, the third hypothesis posits that the split between *roo *and *rooA *corresponds to the split between the genomes of the Sophophora group and the other three genomes. In the Sophophora group the ancestral *roo*/*rooA *element evolved into today's *roo *elements, as indicated in Figure 4c (red lines). In the three genomes of *D. mojavensis*, *D. virilis *and *D. grimshawi *the ancestral element evolved into the *rooA *element. But in contrast to the *roo *elements in the Sophophora group, the *rooA *element did not replicate often and was lost early from *D. virilis *and *D. grimshawi*. Only in *D. mojavensis *did the element start to spread. At one point a *roo *element was horizontally transferred into *D. mojavensis*, and later a second horizontal transfer took place, this time of *rooA *from *D. mojavensis *to the common ancestor of the genomes of the melanogaster subgroup. In this group *rooA *transposed often, especially in the genomes of *D. yakuba *and *D. erecta*. As no functional copy is left in *D. mojavensis*, this horizontal transfer prevented the extinction of the *rooA *element.

In sum, each of these three hypotheses explains some aspect of the available data, but this data is currently not sufficient to decide equivocally for or against one of them.

## Conclusion

Within genomes, the evolutionary dynamics of *roo *and *rooA *shows a broad spectrum ranging from recent intense transpositional activity to slow decay and extinction. Among genomes, the balance of evidence suggests an origin of *roo*/*rooA *in the common ancestor of all 12 genomes or within the *Drosophila *clade, and the separation between the two families shortly afterwards. Furthermore, one horizontal transfer of *roo *and one of *rooA *may have occurred, but the evidence for such transfers is equivocal. Our analysis is based on a limited number of genomes, and conclusive proof of the evolutionary dynamics of *roo *and *rooA*, as well as dating of their origin would need sequence information from additional species. For example, horizontal transfer of *roo *and *rooA *could be confirmed if future genome sequencing showed that all *roo *elements occur in species closely related to the *Sophophora *group and to *D. mojavensis*, whereas *rooA *elements were found to occur only in genomes of the melanogaster subgroup and outside the *Sophophora *group.

## Methods

### Element Identification

To identify *roo *elements in the 12 focal *Drosophila *genomes, we carried out a high stringency and a low stringency search. Both of these searches use an iterative procedure that has as its core the tool IScan[41], which was originally designed to identify insertion sequences, simple transposable elements in bacteria. To identify a transposable element's coding region, IScan performs a tblastn search (using WUBLAST[42]) to identify matches of the amino acid query sequence in a genome sequence of interest. After having identified the coding region, IScan also allows the identification of indirect repeat regions characteristic of some transposable elements, and associated with the coding regions. For our purpose, we modified IScan to allow us to identify the direct repeats associated with *roo *elements.

In our high stringency search, we used the *roo *canonical sequence [GenBank:AY180917] and the *rooA *canonical sequence [18] as a first query, and searched for matches in the 12 *Drosophila *genomes which we obtained from the Comparative Assembly Freeze 1 (CAF1) [43]. In the high stringency search, we considered further only hits with an evalue ≤ 0.0001 and at least 50 percent identical residues between the hit and query sequence. We only considered hits with an LTR sequence length between 100 bp and 1000 bp, and allowed no unidentified ('N') nucleotides in the coding and LTR sequences.

After having identified a series of *roo *sequences that fulfill these criteria, we used these sequences as new queries in a new search round, and reiterated this procedure until no new *roo *sequences were found. Between each iteration, we verified that no previously identified sequence was used more than once as a query. We then manually inspected all *roo *elements for spurious hits.

For the low stringency search, we used those 118 elements previously identified in the high stringency search where the predicted protein length was longer than 1180 aa, that is, at least half of the canonical protein length. For this low stringency search, we used an evalue ≤ 0.01, required at least a 40 percent match between the query sequences and the hit, and permitted sequences with unidentified nucleotides ('N') in their coding and LTR sequences. This search yielded additional *roo *and *rooA *fragments in only one genome (*D. simulans*) where the previous search had failed. However, these elements are of spurious validity, as they contain many sequence fragments annotated with unknown nucleotides ('N'). In all other genomes except *D. persimilis *where the high stringency search had identified one *roo *element, the low stringency search yielded additional *roo *and *rooA *copies. Because both canonical sequences identify *roo *and *rooA *elements we calculated the similarity of each identified element to both canonical elements and added the element to the family with the higher similarity.

### Identification of complete coding region

Our search strategy described above may identify candidate coding regions only incompletely. Specifically, its results may not include the upstream-most and downstream-most parts of coding regions, such as start codons and stop codons. To help us to get complete coding region information, we applied the getorf tool from the EMBOSS package [44] to the complete element sequence, from the first nucleotide of the 5' LTR sequence through the last nucleotide of the 3' LTR.

Specifically, this processing step used the coding region C identified in the initial analysis, and used getorf to identify all open reading frames (ORFs) in the same reading frame as C and overlapping C. For most elements (176 elements), this analysis found only one ORF, which covered C completely, which we then used as the final coding sequence. However, in 54 *roo *and *rooA *elements we found two (or more) ORFs overlapping C, which may be caused by nonsense mutations that insert stop codons into a gene. For these elements, we designated the final coding sequence as extending from the first (upstream-most) to the last (downstream-most) ORF overlapping C. The potential non-coding region between the ORFs, if any, were also included into this final region. With their median length of 270 bp they comprise a small average fraction of 5.6 percent of the coding region, and are thus not likely to significantly alter our results. In fewer than 5 cases additional ORFs in the reading frame but not overlapping C showed a high similarity to the canonical *roo *sequence, in which case we included these ORFs into the coding region as well.

### Sequence alignment and phylogenetic analysis

The alignments we used are based on the protein coding sequences of all *roo *and *rooA *elements. Our dataset contained 230 *roo *and *rooA *elements with coding sequences as long as 2615 amino acids, which renders accurate multiple alignment identification challenging. We compared different alignment algorithms (ClustalW[45], Mafft[46], Muscle[47], Prank[48] and ProbCons[49], T-Coffee[50]) to find the best algorithm for our dataset. Because of its high speed and low number of artefacts evident upon visual inspection, we chose Mafft version 5, which refines alignments iteratively using pairwise alignment information.

Based on the multiple alignment, we computed phylogenetic trees of elements using PhyML_aLRT[27] a version of PhyML[28] which incorporates an approximate likelihood ratio test to estimate the statistical support of the tree topology. This approach is superior to a bootstrap calculation with respect to accuracy and power, and it is computationally much more efficient [27]. The method assigns to each branch a statistical significance ranging from 0 (least significant) to 1 (highly significant). We used the default options of PhyML_aLRT with the JTT matrix for amino acid substitutions, the proportion of invariable sites set to zero, and with only one category of substitution rate [28]. We chose the χ^2^-based parametric branch support for approximate likelihood ratio tests [27]. As outgroup we used the canonical sequence of the *BEL *element in *D. melanogaster *to root the tree.

### Age calculation

We estimate the intra-element pairwise nucleotide identity between LTR sequences using the dnadist program from the PHYLIP package [51] with the Felsenstein F84 parameter method. We used the formula *T *= *K*/2*r *where *T *= time to most recent common ancestry, *K *= sequence divergence, and *r *= substitution rate [23] to calculate the age of an element, as described by Bowen and McDonald (2001) [10]. As a substitution rate we used 0.016 substitutions per site per million years, as estimated for *Drosophila *[23].

### Calculation of amino acid similarity

The calculation is based on the coding region of the element. To avoid biases associated with truncated copies, which are usually also highly diverged, we used only the 134 *roo *and *rooA *elements with an encoded protein sequence length longer than 1180 amino acids, which is half the length of the canonical element's coding region. Using the protdist program from the PHYLIP package [51] we calculated the percent similarity between pairwise Mafft alignments of element pairs.

### Domain identification

The *pol *gene of all autonomous LTR retrotransposons contains a reverse transcriptase (RT), an integrase (IN), and a protease (PR) domain. We obtained the following hidden markov models [52] from Pfam [53]: "RVT_1" for reverse transcriptase, "rve" for integrase, and "Peptidase_A17" for the protease domain. Using Hmmer [54] to search for these domains in our high stringency hits, we then used the identified domains in a tblastn search against all twelve *Drosophila *genomes. Only hits which share at least 99 percent of the length to their query sequence and a sequence identity of 90 percent were considered for further analysis. The amino acid similarity between domains was estimated as described above.

### Search in trace archives

We used the protein sequences of all *roo *and *rooA *elements in a tblastn search against the traces archives of all genomes, except that of *D. pseudoobscura*, where no trace archive was available. A hit required at least 70 percent similarity to a *roo *or *rooA *sequence, and it had to overlap at least half of the trace archive sequence.

## Authors' contributions

NC carried out the research. NC and AW designed the study and wrote the manuscript. All authors read and approved the final manuscript.

## Supplementary Material

Additional file 1 **List of all full length roo and rooA elements**. A list of all 230 full length *roo *and *rooA *elements including species name, accession number, start and end position of the complete element, start and end positions for both LTR sequences and the open reading frame (ORF), the elements age in million years, and the coding strand. The last column indicates whether the element is a *roo *element (0) or a *rooA *element (1). Click here for file

Additional file 2 **LTR sequence distance distribution**. The histograms show the estimated distance between the LTR sequences of *roo *(red bars) and *rooA *(blue bars). Notice the different scales on the y-axis. The vertical red and blue lines indicate the median distance in each genome for *roo *and *rooA*, respectively. Two *roo *elements of *D. mojavensis *had to be excluded because their LTR sequences were falsely identified. The histogram in the bottom right corner shows the distance of all *roo *and *rooA *elements. myr: million years. Click here for file

Additional file 3 **Complete phylogenetic tree of roo and rooA elements**. The complete phylogenetic tree of *roo *and *rooA *elements is shown. Each element is named by the first four letters of their genome and a consecutive number, e. g., Dmel3 is the third element from *D. melanogaster*. *RooA *elements have an additional "A" at the end. The tree is rooted by the *BEL *element, a LTR element from *D. melanogaster*. *Dsim*: *D. simulans*; *Dsec*: *D. sechellia*; *Dmel*: *D. melanogaster*; *Dyak*: *D. yakuba*; *Dere*: *D. erecta*; *Dana*: *D. ananassae*; *Dper*: *D. persimilis*; *Dwil*: *D. willistoni*; *Dmoj*: *D. mojavensis*. Click here for file

Additional file 4 **Average amino acid similarity between reverse transcriptase domains**. All reverse transcriptase domains were used to estimate the amino acid similarity for every pair of these elements using protdist from the PHYLIP package [51]. *Dsim*: *D. simulans*; *Dsec*: *D. sechellia*; *Dmel*: *D. melanogaster*; *Dyak*: *D. yakuba*; *Dere*: *D. erecta*; *Dwil*: *D. willistoni*; *Dmoj*: *D. mojavensis*. Click here for file

Additional file 5 **Average amino acid similarity between integrase domains**. All integrase domains were used to estimate the amino acid similarity for every pair of these elements using protdist from the PHYLIP package [51]. *Dsim*: *D. simulans*; *Dsec*: *D. sechellia*; *Dmel*: *D. melanogaster*; *Dyak*: *D. yakuba*; *Dere*: *D. erecta*; *Dana*: *D. ananassae*; *Dpse*: *D. pseudoobscura*; *Dper*: *D. persimilis*; *Dwil*: *D. willistoni*; *Dmoj*: *D. mojavensis*. Click here for file

Additional file 6 **Average amino acid similarity between the protease domains**. All protease domains were used to estimate the amino acid similarity for every pair of these elements using protdist from the PHYLIP package [51]. *Dsim*: *D. simulans*; *Dsec*: *D. sechellia*; *Dmel*: *D. melanogaster*; *Dyak*: *D. yakuba*; *Dere*: *D. erecta*; *Dana*: *D. ananassae*; *Dwil*: *D. willistoni*; *Dmoj*: *D. mojavensis*. Click here for file
