# Supplementary figures and images for: Evolutionary dynamics of the LTR retrotransposons roo and rooA inferred from twelve complete Drosophila genomes
Source: BMC Evol Biol. 2009 Aug 18;9:205. doi: 10.1186/1471-2148-9-205 (PMC3087523; doi:10.1186/1471-2148-9-205)

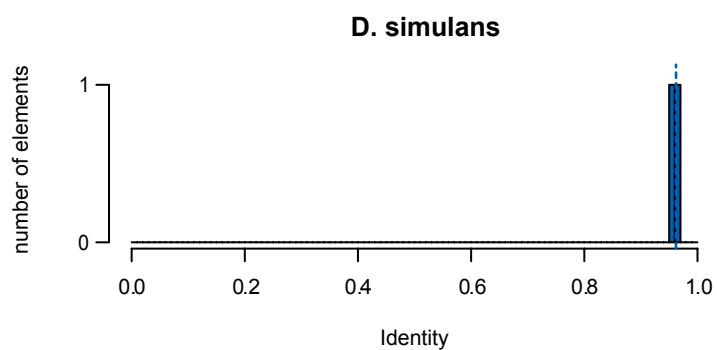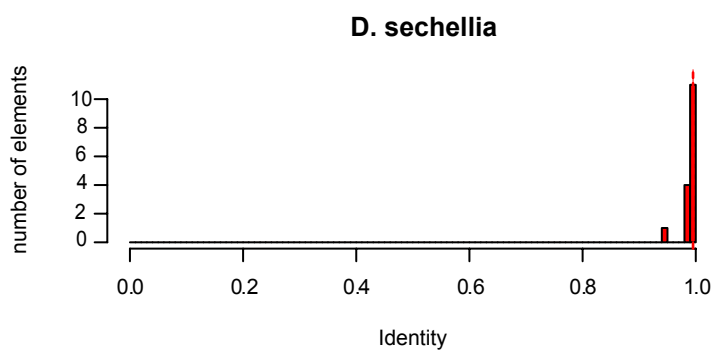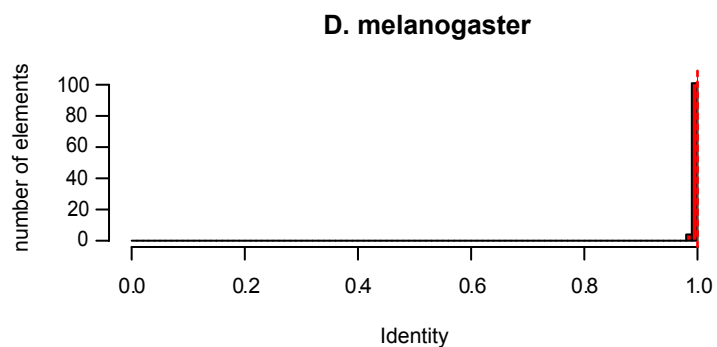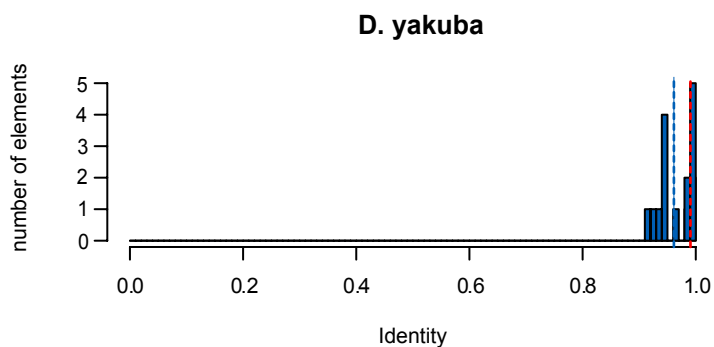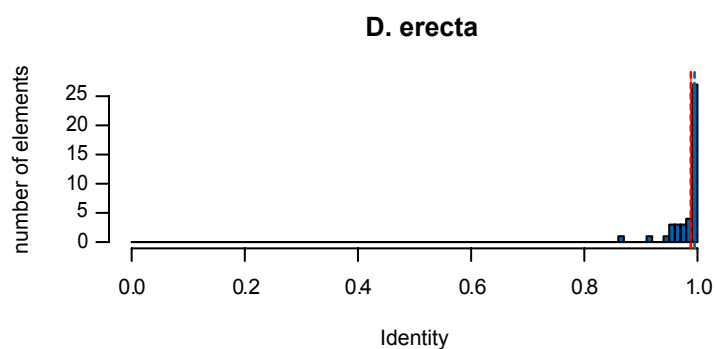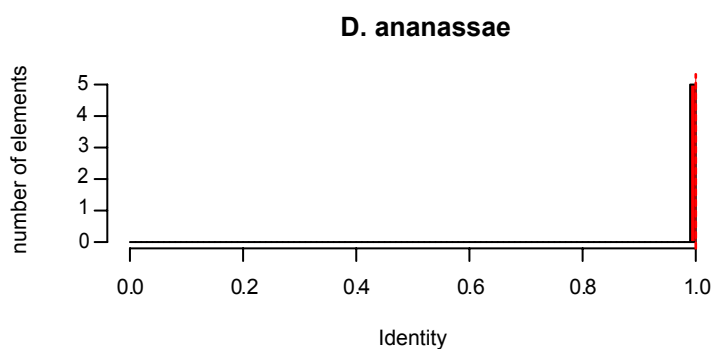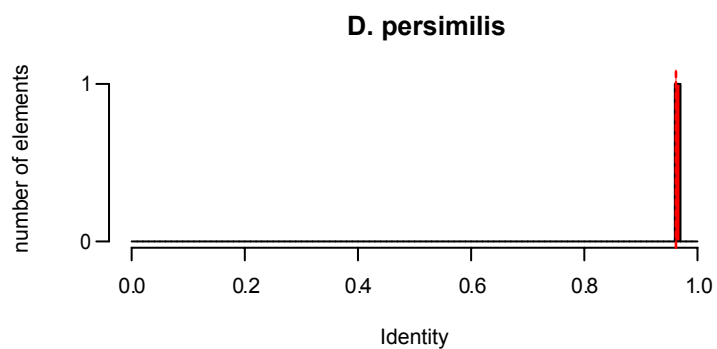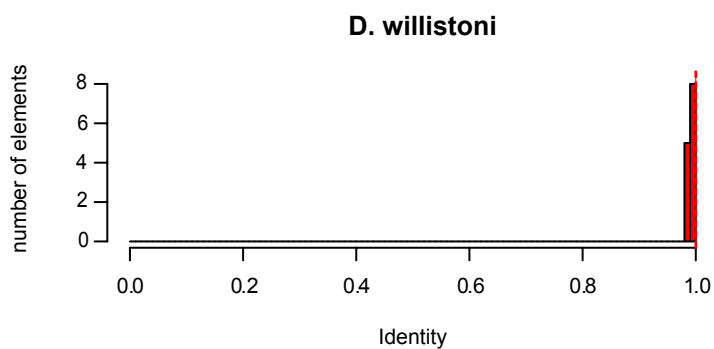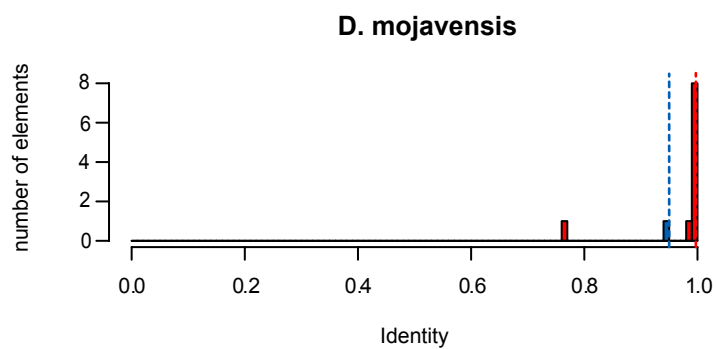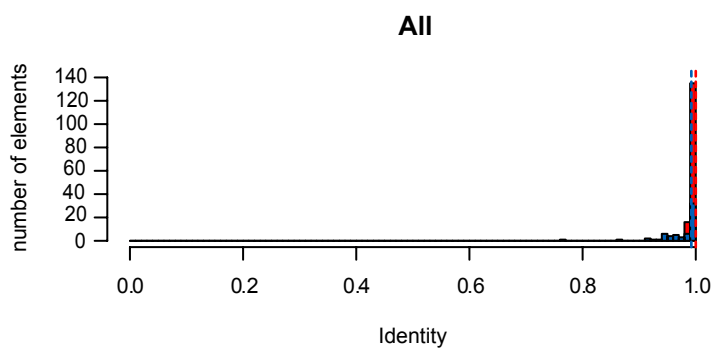

Supplement: Additional file 2 — LTR sequence distance distribution. The histograms show the estimated distance between the LTR sequences of roo (red bars) and rooA (blue bars). Notice the different scales on the y-axis. The vertical red and blue lines indicate the median distance in each genome for roo and rooA, respectively. Two roo elements of D. mojavensis had to be excluded because their LTR sequences were falsely identified. The histogram in the bottom right corner shows the distance of all roo and rooA elements. myr: million years. [file 1471-2148-9-205-S2.pdf]

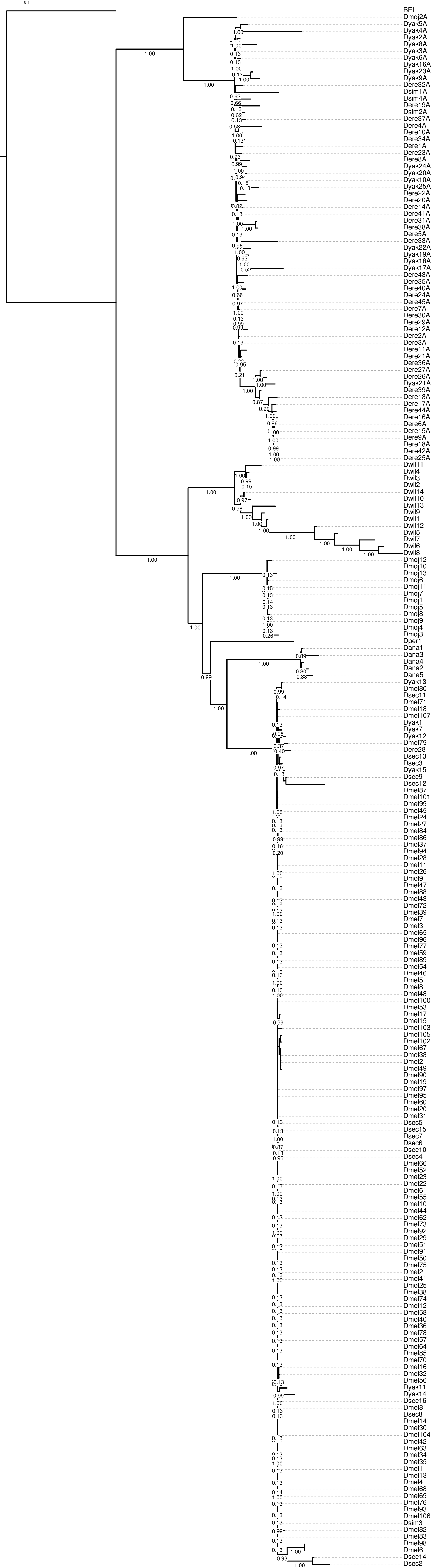

Supplement: Additional file 3 — Complete phylogenetic tree of roo and rooA elements. The complete phylogenetic tree of roo and rooA elements is shown. Each element is named by the first four letters of their genome and a consecutive number, e. g., Dmel3 is the third element from D. melanogaster. RooA elements have an additional "A" at the end. The tree is rooted by the BEL element, a LTR element from D. melanogaster. Dsim: D. simulans; Dsec: D. sechellia; Dmel: D. melanogaster; Dyak: D. yakuba; Dere: D. erecta; Dana: D. ananassae; Dper: D. persimilis; Dwil: D. willistoni; Dmoj: D. mojavensis. [file 1471-2148-9-205-S3.pdf]
