# Supplementary material for: Evolutionary dynamics of the LTR retrotransposons roo and rooA inferred from twelve complete Drosophila genomes
Source: BMC Evol Biol. 2009 Aug 18;9:205. doi: 10.1186/1471-2148-9-205 (PMC3087523; doi:10.1186/1471-2148-9-205)
Supplement: Additional file 5 — Average amino acid similarity between integrase domains. All integrase domains were used to estimate the amino acid similarity for every pair of these elements using protdist from the PHYLIP package [51]. Dsim: D. simulans; Dsec: D. sechellia; Dmel: D. melanogaster; Dyak: D. yakuba; Dere: D. erecta; Dana: D. ananassae; Dpse: D. pseudoobscura; Dper: D. persimilis; Dwil: D. willistoni; Dmoj: D. mojavensis. [file 1471-2148-9-205-S5.pdf]

	roo										*rooA*					
	Dsim	*Dsec*	*Dmel*	*Dyak*	*Dere*	*Dana*	*Dpse*	*Dper*	*Dwil*	*Dmoj*	*Dsim*	*Dsec*	*Dmel*	*Dyak*	*Dere*	*Dmoj*
Dsim	99.7	99.6	99.7	97.6	98.3	79.6	70.9	72.4	74.6	77.7	64.5	65.1	63.9	67.2	67.4	66.0
Dsec		99.6	99.6	97.5	98.2	79.7	71.0	72.5	74.7	77.7	64.7	65.3	63.9	67.4	67.5	66.2
Dmel			99.6	97.6	98.3	79.6	70.9	72.4	74.5	77.6	64.6	65.2	64.0	67.2	67.4	66.1
Dyak				96.6	97.1	78.1	69.7	71.1	73.4	76.7	63.5	64.0	62.8	65.8	66.1	65.3
Dere					100	78.7	61.0	71.5	74.7	76.7	64.2	64.7	63.5	66.9	67.0	65.6
Dana						97.5	73.7	76.3	77.9	77.9	66.0	67.3	65.2	68.2	68.2	69.7
Dpse							89.5	92.4	76.6	72.6	63.3	65.8	62.1	64.9	65.9	66.7
Dper								94.7	78.4	74.1	64.7	66.0	63.7	66.3	67.3	68.3
Dwil									96.5	76.9	63.7	64.8	62.5	66.2	66.4	66.0
Dmoj										98.6	66.1	67.5	63.9	68.0	68.3	69.1
Dsim											89.9	91.4	88.8	90.5	93.0	79.2
Dsec												100	90.5	91.9	94.1	80.4
Dmel													100	81.0	92.0	78.3
Dyak														93.2	94.6	80.0
Dere															97.2	81.4
Dmoj																98.0
