# Supplementary material for: Evolutionary dynamics of the LTR retrotransposons roo and rooA inferred from twelve complete Drosophila genomes
Source: BMC Evol Biol. 2009 Aug 18;9:205. doi: 10.1186/1471-2148-9-205 (PMC3087523; doi:10.1186/1471-2148-9-205)
Supplement: Additional file 6 — Average amino acid similarity between the protease domains. All protease domains were used to estimate the amino acid similarity for every pair of these elements using protdist from the PHYLIP package [51]. Dsim: D. simulans; Dsec: D. sechellia; Dmel: D. melanogaster; Dyak: D. yakuba; Dere: D. erecta; Dana: D. ananassae; Dwil: D. willistoni; Dmoj: D. mojavensis. [file 1471-2148-9-205-S6.pdf]

	roo								*rooA*				
	Dsim	*Dsec*	*Dmel*	*Dyak*	*Dere*	*Dana*	*Dwil*	*Dmoj*	*Dsim*	*Dsec*	*Dmel*	*Dyak*	*Dere*
Dsim	99.7	99.8	99.7	98.8	98.1	70.5	67.0	65.2	57.6	55.9	51.2	56.2	56.2
Dsec		99.8	99.8	98.8	98.1	70.6	67.1	65.3	57.7	56.0	54.6	56.3	56.3
Dmel			99.7	98.7	98.0	70.5	67.1	65.3	57.6	56.0	54.5	56.3	56.3
Dyak				97.9	97.3	69.7	66.4	64.7	57.0	55.2	53.9	55.5	55.6
Dere					96.9	67.6	63.2	61.2	54.3	52.4	51.2	52.9	52.9
Dana						98.3	62.7	64.3	59.9	58.4	56.3	58.3	58.5
Dwil							92.3	66.0	56.2	54.2	54.2	54.8	54.7
Dmoj								97.8	57.4	55.1	53.5	55.5	55.7
Dsim									95.9	95.0	91.6	95.6	94.9
Dsec										94.7	89.9	93.9	93.3
Dmel											99.3	92.1	91.6
Dyak												96.1	95.5
Dere													96.5
